# Signatures of enhanced out-of-plane polarization in asymmetric BaTiO_3_ superlattices integrated on silicon

**DOI:** 10.1038/s41467-021-27898-x

**Published:** 2022-01-11

**Authors:** Binbin Chen, Nicolas Gauquelin, Nives Strkalj, Sizhao Huang, Ufuk Halisdemir, Minh Duc Nguyen, Daen Jannis, Martin F. Sarott, Felix Eltes, Stefan Abel, Matjaž Spreitzer, Manfred Fiebig, Morgan Trassin, Jean Fompeyrine, Johan Verbeeck, Mark Huijben, Guus Rijnders, Gertjan Koster

**Affiliations:** 1 MESA+ Institute for Nanotechnology, University of Twente, 7500 AE Enschede, The Netherlands; 2 Key Laboratory of Polar Materials and Devices (MOE) and Department of Electronics, East China Normal University, Shanghai, 200241 China; 3 Electron Microscopy for Materials Science (EMAT), University of Antwerp, 2020 Antwerp, Belgium; 4 Department of Materials, ETH Zurich, Vladimir-Prelog-Weg 4, 8093 Zurich, Switzerland; 5 Lumiphase AG, 8802 Kilchberg, Switzerland; 6 Advanced Materials Department, Jožef Stefan Institute, 1000 Ljubljana, Slovenia

**Keywords:** Ferroelectrics and multiferroics, Surfaces, interfaces and thin films

## Abstract

In order to bring the diverse functionalities of transition metal oxides into modern electronics, it is imperative to integrate oxide films with controllable properties onto the silicon platform. Here, we present asymmetric LaMnO_3_/BaTiO_3_/SrTiO_3_ superlattices fabricated on silicon with layer thickness control at the unit-cell level. By harnessing the coherent strain between the constituent layers, we overcome the biaxial thermal tension from silicon and stabilize *c*-axis oriented BaTiO_3_ layers with substantially enhanced tetragonality, as revealed by atomically resolved scanning transmission electron microscopy. Optical second harmonic generation measurements signify a predominant out-of-plane polarized state with strongly enhanced net polarization in the tricolor superlattices, as compared to the BaTiO_3_ single film and conventional BaTiO_3_/SrTiO_3_ superlattice grown on silicon. Meanwhile, this coherent strain in turn suppresses the magnetism of LaMnO_3_ as the thickness of BaTiO_3_ increases. Our study raises the prospect of designing artificial oxide superlattices on silicon with tailored functionalities.

## Abstract (web summary)

Integrating multifunctional oxides on silicon is highly desirable. Here, the authors present asymmetric BaTiO3 superlattices on silicon exhibiting enhanced out-of-plane polarization by harnessing the interfacial strain and broken inversion symmetry.

## Introduction

Ultrathin ferroelectrics are key components in ferroelectric field effect transistors and ferroelectric tunnel junctions, holding great promise for applications in high-density non-volatile memories and logic devices^1^. However, it remains challenging to integrate ferroelectric thin films (perovskite ferroelectrics, for example) with desired out-of-plane polarization onto the silicon platform. The structural difference and chemical incompatibility largely hinder the epitaxial growth of ferroelectric oxides directly on silicon^2^. The SrTiO_3_ (STO) buffer layer prepared by molecular beam epitaxy (MBE) has been widely used as a template to overcome this limitation, but the defective STO layer usually induces a relaxation of the crystalline film at the growth temperature, and the typically large thermal-expansion coefficients in oxides leads to biaxial thermal tension to the ferroelectric film, favouring an in-plane polarization^3,4^. On the other hand, most ferroelectric thin films suffer from the finite size effect, i.e., the out-of-plane ferroelectricity degrades and eventually disappears when scaling down the film thickness to few nanometers^5–8^. Such effect has been ascribed to the increasing depolarization field in thinner films arising from incomplete screening of bound charges at the ferroelectric/metal interfaces^9^.

By contrast to the size effect in ferroelectric single films, ferroelectricity can be retained or even enhanced at reduced dimensions in ferroelectric superlattices (SLs) via engineering the mechanical and electrical boundary conditions^10–16^. Ferroelectricity has been probed in BaTiO_3_ (BTO)/STO SLs with one-unit-cell (uc)-thick BTO^10^. Upon increasing the volume fraction of BTO, robust ferroelectricity and enhanced transition temperature can be realized because of the compressive strain in conjunction with the highly polarizable nature of the incipient ferroelectric STO^10–14^. A distinct scenario has been proposed for short-period PbTiO_3_ (PTO)/STO SLs where the strain effect is minimal^15,16^. The competition between the antiferrodistortive rotations of TiO_6_ octahedra and ferroelectric displacement gives rise to a restored improper ferroelectricity when PTO layer is thinner than 3-uc^15,16^. In particular, it has been theoretically predicted and experimentally verified that ferroelectricity can be enhanced in three-component SLs due to the broken inversion symmetry^17–19^. The dipoles created at the interfaces can accumulate with repeating the stackings, yielding a macroscopic polarization even without involving any ferroelectric components^20,21^. Aside from the enhancement of ferroelectricity, negative capacitance, polar vortices and polar skyrmions have been recently demonstrated in ferroelectric SLs^22–24^. In view of the enhanced properties along with the discoveries in ferroelectric SLs, it would be of practical interest to integrate them onto the technologically important silicon substrates.

In this work, LaMnO_3_ (LMO)/BTO/STO SLs are formed on silicon with a MBE-grown STO buffer layer. Here LMO is intentionally introduced to break the symmetry of the environment of the BTO layer, which is expected to enhance its polarization^17–19^. Meanwhile, the SL consisting of ferroelectric and magnetic materials can be a potential multiferroic system for electric-field controlled magnetism^25^. Atomically resolved scanning transmission electron microscopy (STEM) reveals *c*-axis-oriented BTO layers with remarkably enhanced tetragonality (*c*/*a*). This is accomplished by taking advantages of the compressive strain from neighboring STO and LMO layers, which overcomes the biaxial thermal tension from silicon. Optical second harmonic generation (SHG) measurements show signatures of a dominant out-of-plane polarized state in the SLs with strongly enhanced polarization, as compared to the BTO film and BTO/STO SL grown on silicon. On the other hand, this coherent strain is manifested by a gradually suppressed magnetism of LMO upon increasing the layer thickness of BTO.

## Results

### Fabrication of LMO/BTO/STO SLs

**Fig. 1:**
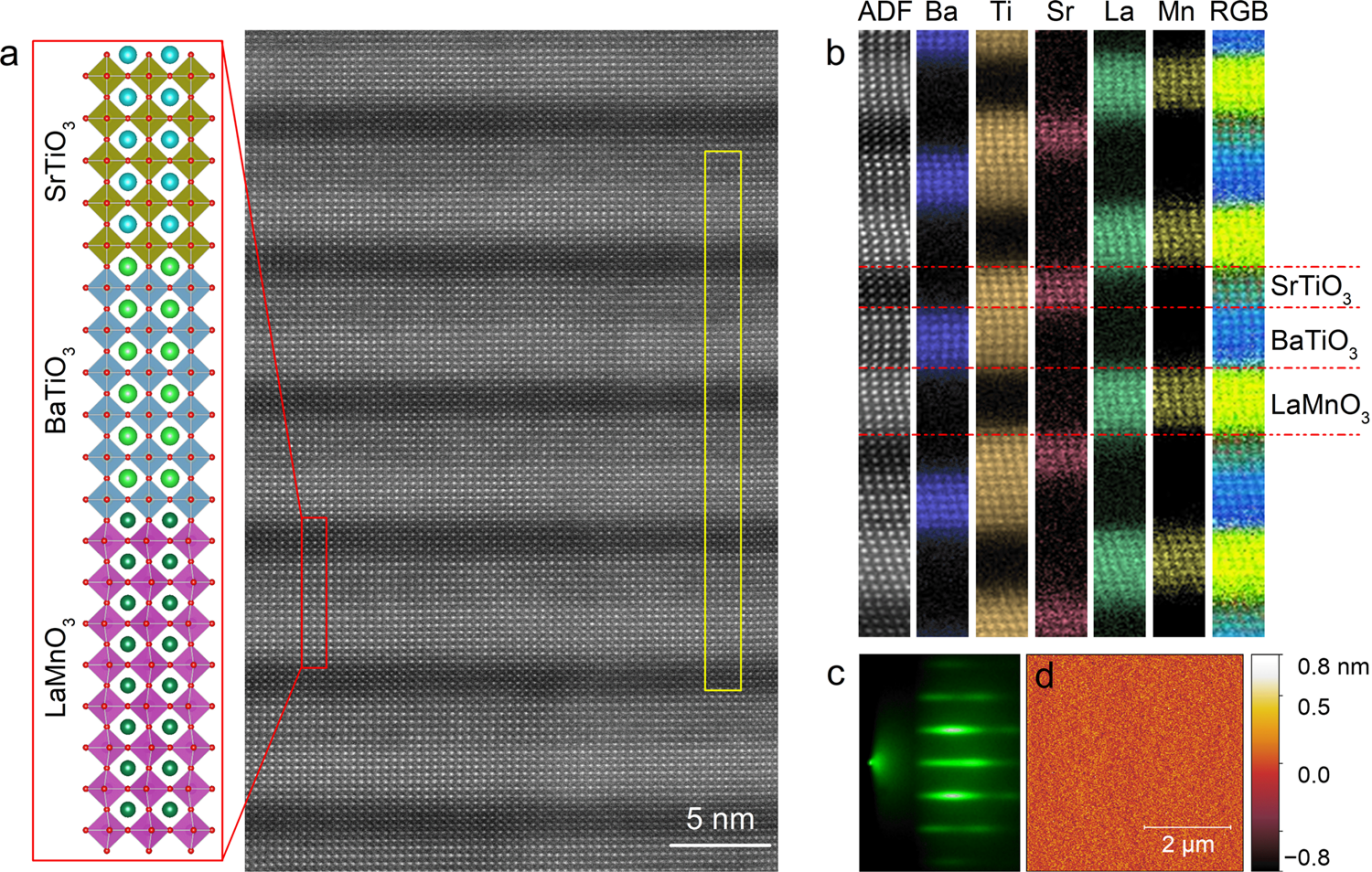
Fabrication of LMO/BTO/STO SLs on Si/STO. **a** HAADF-STEM image of the L_8_B_6_S_5_ SL grown on Si/STO. The sketch on the left shows the ideal atomic structure in one period. The EELS elemental maps taken from the yellow rectangular area are shown in (**b**). **c**, **d** Show the RHEED image taken along the Si[110] azimuth and AFM image of the L_8_B_6_S_5_ SL.

We fabricated LMO/BTO/STO SLs on STO-buffered silicon substrates using pulsed laser deposition (PLD) with in situ reflection high-energy electron diffraction (RHEED). The 4-nm thick STO buffer layer was grown by MBE and the growth details can be found elsewhere^26^. Ferroelectric BTO is tetragonal at room temperature with *a* = *b* = 3.992 Å and *c* = 4.036 Å^27^. Cubic STO (*a* = 3.905 Å) is described as an incipient ferroelectric, with ferroelectric transition suppressed by quantum fluctuations^28^. Orthorhombic LMO is an A-type antiferromagnetic insulator in its stoichiometric bulk, but usually turns into a ferromagnetic insulator in its thin-film form^29^. The lattice constants depend on its oxygen content, and the strain-free LMO grown in this study has a lattice constant *a*_pc_ ≈ 3.915 Å (pc, pseudocubic)^30,31^. Therefore, if the SLs are fully relaxed from silicon while the lattice coherency in between the component layers is preserved, BTO will receive compressive strain from the adjacent STO and LMO, which can be utilized to compensate the thermal tension from silicon and enhance the out-of-plane ferroelectricity of BTO^27^. In this work, the SLs are formed by repeating LMO/BTO/STO trilayer 10 times, where the layer thicknesses of LMO and STO are fixed to 8-uc and 5-uc, and the layer thickness of BTO (*n*) is varied from 0 to 12 uc. The corresponding SL is abbreviated as L_8_B_*n*_S_5_ hereafter. All the three components show a quasi two-dimensional layer-by-layer growth mode throughout the entire deposition (Supplementary Fig. 1), enabling us to design the SL composition with uc accuracy. Figure 1a displays the high-angle annular dark filed (HAADF)-STEM image of the L_8_B_6_S_5_ SL, with the ideal atomic structure in one period schematically drawn on the left. The well-defined SL structure is formed with commensurate interfaces, although the contrast between LMO and BTO layers is not sharp because of the very close atomic numbers of La and Ba. The chemically abrupt interfaces are further corroborated by electron energy loss spectroscopy (EELS) elemental mappings as shown in Fig. 1b and Supplementary Fig. 2. Importantly, STEM characterizations confirm the perfect lattice coherency in between the component layers in spite of the large lattice mismatch (~2%) between BTO and STO (LMO), which is crucial to maintain a compressively strained BTO with *c*-axis orientation. Previous STEM study revealed a critical thickness of 5 nm, above which misfit dislocations appear, for BTO single films grown on the STO-buffered Si substrates^32^. Here the preserved strain state over a much greater overall thickness in the SL geometry offers more opportunities to strain engineering in epitaxial systems, especially those with large lattice mismatch^12,19,33,34^. Figure 1c shows the streaky RHEED pattern of the L_8_B_6_S_5_ SL, indicative of a smooth surface. The atomic force microscopy (AFM) image shown in Fig. 1d reveals a root-mean-square roughness of ~1.3 Å (see more AFM images in Supplementary Fig. 3), which is comparable to the underlying 4-nm STO buffer layer grown by MBE.

**Fig. 2:**
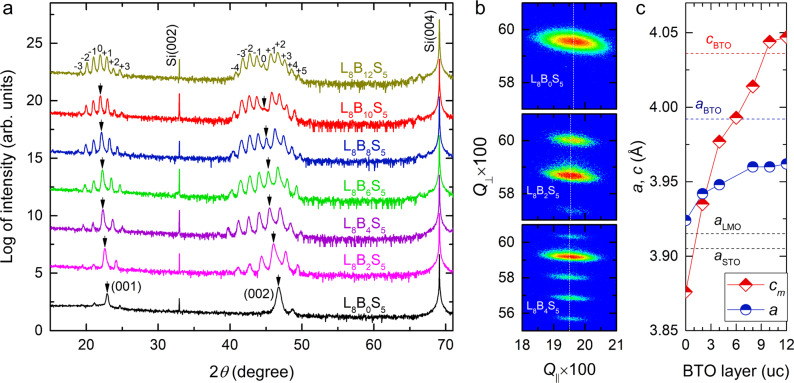
Thickness dependent strain states in LMO/BTO/STO SLs. **a** XRD *θ*-2*θ* scans of the SLs with varied layer thickness of BTO grown on Si/STO. The arrows indicate the (001) and (002) main peaks. The satellite peaks are indexed for the L_8_B_12_S_5_ SL. **b** RSMs around the (103)_pc_ reflection of L_8_B_0_S_5_, L_8_B_2_S_5_ and L_8_B_4_S_5_. **c** Extracted in-plane (mean out-of-plane) lattice constant, *a* (*c*_*m*_) as a function of the layer number of BTO. The dashed lines indicate the lattice constants of bulk STO, LMO and BTO.

Figure 2a depicts high-resolution X-ray diffraction (XRD) linear scans of LMO/BTO/STO SLs with varied *n*. Multiple satellite peaks are observed around the (001) and (002) main Bragg reflections, corroborating the well-defined artificial periodicity of the SL structures. The periodic lengths derived from those satellite peaks are in excellent agreement with our design. The main peaks indicated by arrows gradually shift to lower angles upon increasing *n* due to the much greater out-of-plane lattice constants of BTO than STO and LMO. The gradually weakened intensity of the (002) main peak for *n* ≥ 6 is in close resemblance to the BTO/STO SLs grown on STO substrates^14^. The gradual extinction of the (002) peak can be well captured by XRD simulations without involving extra thickness fluctuations or strain relaxations (Supplementary Fig. 4), meaning that this trend is intrinsic to the SL series and not correlated with the film quality. The crystallinity was assessed by measuring XRD rocking curves around the SL (001) peak (Supplementary Fig. 5a). A typical full width at half maximum of ~0.8° is obtained. XRD phi scans around Si (202) and SL (101)_pc_ reflections ascertain the cube-on-cube epitaxial relationship between the SL and Si (Supplementary Fig. 5b), with SL [100]_pc_//Si [110]. In order to gain insights into the strain states, we conducted reciprocal space mapping (RSM) around the SL (103)_pc_ reflection as depicted in Fig. 2b and Supplementary Fig. 6. The broad feature is caused by the in-plane mosaic spread of the SLs, which is generally present in oxide films grown on silicon^31,35–37^. As *n* increases, the reflection shifts to smaller in-plane *Q* value, corresponding to an expanded in-plane lattice spacing. This means the compressive strain applied to BTO decreases with increasing its volume fraction. The derived in-plane lattice constant (*a*) along with the mean out-of-plane lattice constant (*c*_*m*_) are summarized in Fig. 2c. The LMO/STO SL (L_8_B_0_S_5_) shows *a* = 3.924 Å and *c*_*m*_ = 3.876 Å, pointing to an in-plane tensile strain. This strain is caused by the difference in thermal expansion coefficients between silicon (~2.6 × 10^−6 ^K^−1^) and oxides (~8.7 × 10^−6 ^K^−1^ for STO and ~11.2 × 10^−6 ^K^−1^ for LMO)^31,35^. At the growth temperature, the SLs are fully relaxed from silicon and *a* is mostly determined by the relative thickness ratio among the constituent layers^37^. As the samples cool down to room temperature, *a* is constrained by the additional thermal tension from silicon^31,35,36^. As seen in Fig. 2c, *a* steadily increases from 3.942 Å at *n* = 2 to 3.960 Å at *n* = 8, and then barely changes with further increasing *n*. By assuming that the BTO layers in all SLs are *c*-axis oriented, the in-plane compressive strain applied to BTO can be estimated as −1.3% for *n* = 2 and −0.8% for *n* ≥ 8.

### STEM mapping of tetragonality

**Fig. 3:**
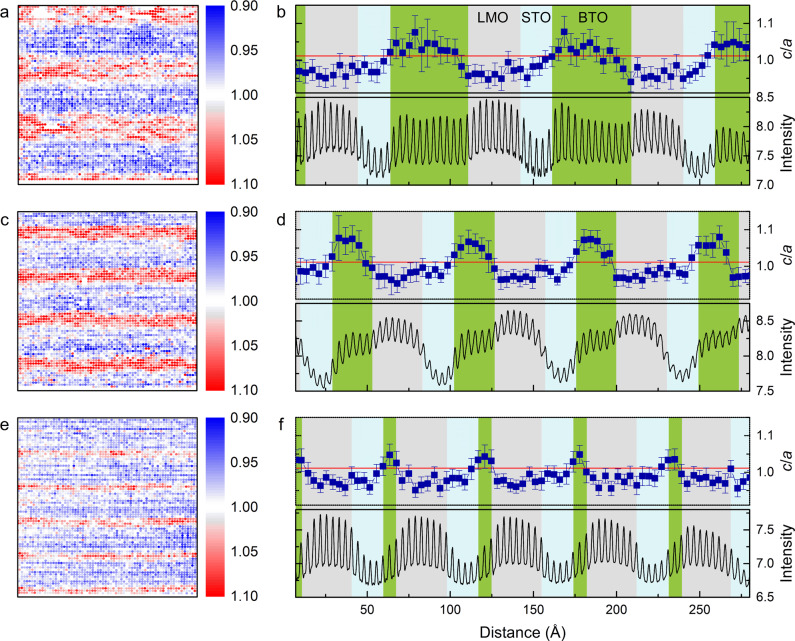
STEM mapping of tetragonality (*c*/*a*). Two-dimensional mapping of *c*/*a* for L_8_B_12_S_5_ (**a**), L_8_B_6_S_5_ (**c**) and L_8_B_2_S_5_ (**e**). The corresponding *c*/*a* averaged within each atomic layer is plotted in (**b**), (**d**) and (**f**), respectively. The error bars show the standard deviation for each atomic plane. In (**b**), (**d**) and (**f**), the red line in upper panels indicates the *c*/*a* value of BTO single crystal, and the bottom panels show the HAADF intensity in arbitrary units for ease of identifying each layer.

The inherently small ionic displacements in BTO and less perfect crystallinity of our samples on silicon pose great challenges to a direct mapping of the dipole moments in the SLs using STEM (see Supplementary Fig. 7 and Fig. 8). In view of the strong strain-polarization coupling in ferroelectrics like BTO and PTO^38^, the evolution of tetragonality (*c/a*) has often been used to trace the changes of polarization in ferroelectric thin films and SLs^11,15,38,39^. Here atomically resolved HAADF-STEM was employed to map out the *c*/*a* of LMO/BTO/STO SLs as displayed in Fig. 3. We measured *a* and *c* from the positions of La, Sr and Ba atoms, which are determined by fitting the HAADF-STEM image to a sum of gaussians using Atomap^40^. Note that LMO is treated as a tetragonal structure in the SL due to the epitaxial constraints from adjacent STO and BTO layers^41^. Figure 3a, c and e shows a 28 × 28 nm^2^ cross-sectional mapping of *c*/*a* for the LMO/BTO/STO SLs with *n* equal to 12, 6 and 2, respectively. The contrast between BTO and LMO (STO) layers is evident. The tensile coherent strain from BTO together with the thermal tension from silicon substrate yield *c*/*a* < 1 for STO and LMO, while the dominant compressive strain from LMO and STO yields *c*/*a* > 1 for BTO. The tetragonality of each atomic plane was averaged along the film normal and plotted as a function of layer distance in Fig. 3b, d, and f. For ease of identifying individual layers in the SLs, we show the layer-resolved HAADF intensity in the corresponding lower panels, where the intensity decreases from LMO to BTO, and then to STO. The red line indicates the *c/a* ~ 1.01 of BTO single crystal. It is found that the L_8_B_12_S_5_ SL shows an average *c/a* ~ 1.03, slightly enhanced than bulk BTO. For the L_8_B_6_S_5_ SL, the enhanced in-plane compressive strain leads to a larger *c/a* ~ 1.06, which is comparable to the strong ferroelectric PTO showing *c/a* ~ 1.06^39^. Surprisingly, the L_8_B_2_S_5_ SL shows a reduced *c/a* ~ 1.04 in spite of the largest in-plane compressive strain. Such a reduction of tetragonality has also been observed in PTO thin films and PTO/STO SLs when scaling down the ferroelectric layer thickness^15,39^. This was interpreted being a consequence of suppressed ferroelectricity in ultrathin PTO layers caused by the unscreened depolarization field. Meanwhile, it has been argued that the weakened ferroelectricity in ultrathin films is related to the interfacial and surface layers having different coordination environments than their bulk counterparts^19,42^. As reported in STO/BTO/CaTiO_3_ SLs grown on STO substrates, the remanent polarization drastically decreases upon reducing the BTO layer thickness from 4 to 2 uc, although they are under the same amount of strain^19^. The results are consistent with our observation of a decreased tetragonality in the L_8_B_2_S_5_ SL where no bulklike BTO is present. Aside from the direct mapping of *c/a*, the tetragonal distortions in BTO layers are also reflected from the changes in the energy splitting of Ti 3*d* levels, as revealed by EELS of Ti L_2,3_ edges (Supplementary Fig. 9). In comparison with the STO layers, BTO shows a relatively smaller splitting energy for the *t*_2g_ and *e*_g_ levels, consistent with the observations in ferroelectric PTO/STO SLs^43^.

### Optical SHG measurements

**Fig. 4:**
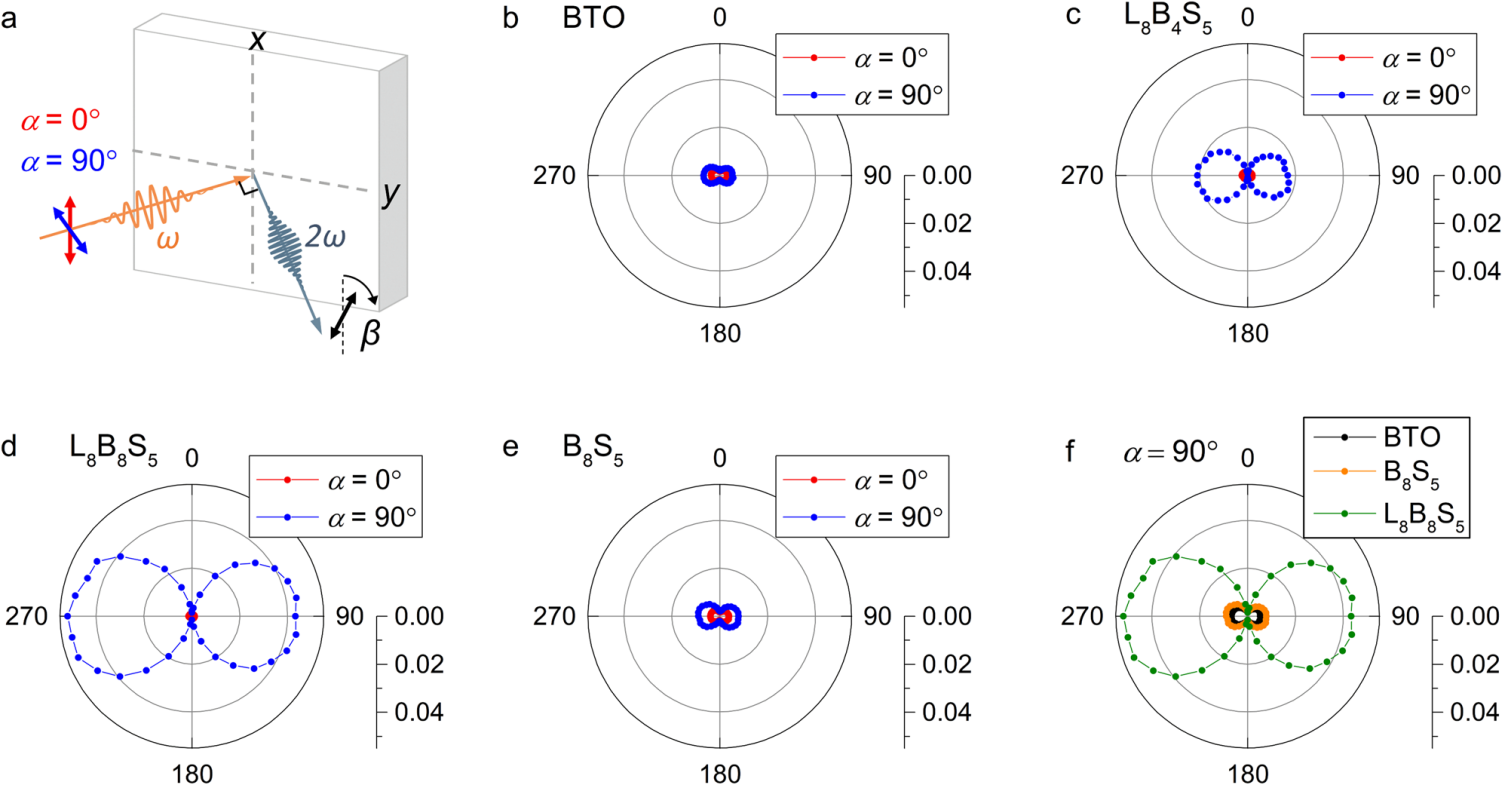
Optical SHG characterizations of LMO/BTO/STO SLs. **a** Schematic of the SHG setup. **b**–**e** SHG analyzer measurements for the 40-uc BTO single film, L_8_B_4_S_5_, L_8_B_8_S_5_ and B_8_S_5_ SLs grown on Si/STO measured with a polarizer angle *α* = 0° and *α* = 90°. **f** SHG signal at a fixed polarizer angle of *α* = 90° comparing the 40-uc BTO, the bicolor B_8_S_5_ SL, and the tricolor L_8_B_8_S_5_ SL.

Characterizing the ferroelectric properties of the SLs using direct hysteresis measurement or piezoresponse force microscopy is restricted due to the lack of appropriate oxide electrodes that can be grown on silicon with sufficiently smooth surface^44–46^. Here optical SHG measurements were carried out to gain insights into the polar nature of the SLs^47^. SHG, the doubling of the frequency of a light wave in a material, is allowed in non-centrosymmetric environments and thus ideal for probing the inversion symmetry-breaking ferroelectric polarization. The SHG setup is used in a reflection geometry as sketched in Fig. 4a. For the 4*mm* point-group symmetry that characterizes the BTO unit cell, one can access the in-plane or the out-of-plane polarization by selecting the polarization angle of the incident *(α*) and the generated light (*β*) with respect to the normal to the reflection plane. For instance, the configuration with *α* = *β* = 0° probes only the in-plane polarization, changing *α* to 90° allows the detection of both the in-plane and out-of-plane polarizations^48^. We monitored the angular dependence of the SHG emitted light (*β*) for a fixed *α* = 0° and 90°. The SHG measurements were carried out at room temperature, far above the LMO magnetic *T*_C_ of all SLs as we will show later, so that a SHG signal of magnetic origin does not have to be considered^49^. To ensure a quantitative comparison of the SHG yield of different samples, the equivalent alignment of each sample is confirmed by a common reference SHG signal that is maximized to the same value on each sample and then filtered out for the measurement itself. In Fig. 4b–d, we compared the SHG responses of a 40-uc BTO single film, L_8_B_4_S_5_ and L_8_B_8_S_5_ SLs. For all three samples, hardly no SHG response is detected for *α* = 0°, indicative of a near zero net in-plane polarization. For the BTO single film, an XRD scan indicates the coexistence of *a*- and *c*- domains (Supplementary Fig. 10), thus the absence of SHG signal for *α* = 0 is mostly likely due to the cancellation of contributions from in-plane *a*- domains with antiparallel polarizations in BTO^48^. In the following, the SHG response for *α* = 90° can be entirely related to the out-of-plane polarization because of the lack of net in-plane polarization for all samples. In contrast to the rather weak SHG signal of the BTO single film, the L_8_B_4_S_5_ SL shows a strong response for *α* = 90° despite the same volume of BTO, strongly supporting the presence of a strain-enhanced out-of-plane polarization in the SL structure. The L_8_B_8_S_5_ SL shows a similar SHG signal with a double-lobe feature, which can be well fitted considering a *4mm* point group (see Supplementary Fig. 11). The SHG intensity at *β* = 90° and 270° is ~2.5 times larger than that of the the L_8_B_4_S_5_ SL. Since the SHG intensity scales with the square of the net polarization and the square of the overall thickness of the ferroelectric material^47^, the results suggest that the polarization of the L_8_B_8_S_5_ SL is weakened as compared to the L_8_B_4_S_5_ SL. This agrees with the reduction of the interfacial compressive strain and tetragonality upon increasing *n*. To check the validity of our SHG measurements, we further performed SHG polarizer scans for the L_8_B_8_S_5_ SL (see Supplementary Fig. 12). The observed SHG signal with fourfold symmetry at *β* = 0° is in perfect agreement with previous studies on BTO single crystal^50^. Moreover, we confirmed the reproducibility of the observed evolution of the SHG yield on our samples with an additional series of SHG measurements, where lower laser fluence and longer acquisition time were used (Supplementary Fig. 13). Figure 4e shows the SHG yield of the conventional bicolor B_8_S_5_ SL. The maximum intensity at *β* = 90° and 270° of the B_8_S_5_ SL is ~5 times smaller than that of the L_8_B_8_S_5_ SL. Note that the numerous interfaces in the B_8_S_5_ SL do not result in a noticeable increase of the total SHG yield in comparison with the single BTO layer (Fig. 4f). Thus, the increased number of interfaces in the tricolor SL cannot explain the drastic enhancement of the SHG response in comparison with the bicolor structure. Given the fact that the compressive strain applied to BTO is slightly smaller in B_8_S_5_ (*a* = 3.968 Å) as compared to L_8_B_8_S_5_ (*a* = 3.960 Å) (Supplementary Fig. 14), the result points to the important role of the inversion symmetry breaking in enhancing the net polarization of the tricolor SLs^49^. It should be noted that our SHG measurements only confirm the presence of polar displacements in the SLs, further studies on the switching properties of these dipoles are needed.

### Strain effects on the magnetism of LMO

**Fig. 5:**
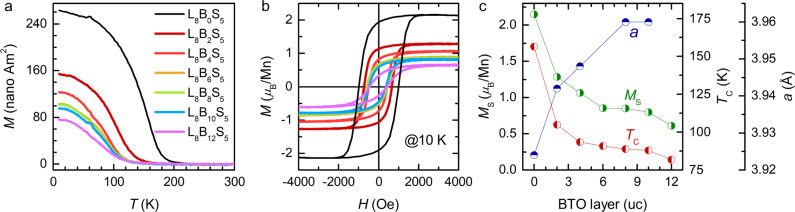
Strain effects on the magnetism. **a**, **b** Temperature dependent magnetization curves and magnetic hysteresis loops at 10 K measured for LMO/BTO/STO SLs with varied layer thickness of BTO. **c** Plots of *M*_S_, *T*_C_ and in-plane lattice parameter (*a*) as a function of the layer number of BTO.

So far we have demonstrated that the compressive strain from LMO and STO layers can promote *c*-axis oriented BTO with enhanced tetragonality in LMO/BTO/STO SLs grown on silicon. On the other hand, the tensile strain from BTO can in turn affect the ferromagnetism of LMO. Figure 5a and b shows the temperature dependent magnetization curves and magnetic hysteresis loops at 10 K for a series of LMO/BTO/STO SLs with varied *n*. The extracted Curie temperature (*T*_C_) and saturation magnetization (*M*_S_) are plotted against *n* in Fig. 5c. The referenced sample, LMO/STO (L_8_B_0_S_5_) SL, shows a relatively high *T*_C_ ~ 156 K and large *M*_S_ ~ 2.15 *μ*_B_/Mn. As BTO layers are interleaved, *T*_C_ and *M*_S_ drastically decrease to 104 K and 1.28 *μ*_B_/Mn at *n* = 2, followed by a gradual decay with further increasing *n*. The suppression of ferromagnetism is in line with the increasing *a* (that is, a measure of the in-plane tensile strain) as shown in Fig. 5c. This can be understood by the tensile-strain-induced antiferromagnetic instabilities in LMO, through promoting an in-plane orbital order and/or enhancing the Jahn-Teller distortions^51,52^. Note that no substantial changes of Mn L_2,3_ edges are observed upon approaching the LMO/BTO interface (Supplementary Fig. 15), thus the weakened magnetism here is unlikely caused by the electrostatic doping effect from BTO^25^. These results point to the intimate correlation between the interfacial strain and magnetism of LMO.

## Discussion

To conclude, by taking advantage of the interfacial compressive strain from LMO and STO layers, we overcome the inevitable thermal tension and stabilize a *c*-oriented BTO with strongly enhanced tetragonality on silicon substrates. In general, the control over the tetragonality and phase transitions in BTO has very important technological implications^53^. Also, the ability to form oxide SLs on silicon with precise layer control is rather encouraging in light of the intriguing phenomena recently uncovered in oxide SLs^54^. The successful preparation of a proper bottom electrode for those SLs will open up more opportunities for the development of electric-field controlled magnetism and ferroelectric photovoltaics^25,55^. Particularly, in a tricolor geometry, asymmetric interface effects can be realized at the bottom and top interfaces of a functional layer, which may lead to improved or emergent properties inaccessible in conventional bicolor SLs^21,56,57^. Further, the integration of high-quality LMO/BTO/STO SLs on silicon indicates several possible avenues for future exploitations, such as strain-controlled formation of polar topologies^23,24^, interface-induced multiferroism^33^, as well as the low-temperature magneto-optical effects endowed by the simultaneously broken time-reversal and spatial-inversion symmetries^49^.

## Methods

### Sample growth and basic characterization

The LMO/BTO/STO SLs were fabricated using PLD with a KrF excimer laser at 248 nm. The laser fluence and repetition rate were set to 2 J/cm^2^ and 2 Hz, respectively. The substrate temperature was held at 650 °C. The depositions were conducted at a relatively low oxygen pressure of 0.01 mbar, at which all the three components can grow with a quasi two dimensional fashion while LMO can retain its ferromagnetic character. After the depositions, the samples were in-situ annealed for 10 min to improve the crystallinity before cooling down to room temperature at a rate of 15 K/min. The X-ray diffractions were performed on PANalytical-X’Pert materials research diffractometer at the high-resolution mode. The surface morphology was characterized using Bruker atomic force microscopy. The magnetic properties were measured using vibrating sample magnetometry on a Quantum Design physical property measurement system.

### STEM/EELS

The characterization of the atomic structure was conducted using Cs-corrected scanning transmission electron microscopy high angle dark field imaging (STEM-HAADF) on the X-Ant-Em instrument at the University of Antwerp operated at 300 kV, a convergence angle of 20 mrad and a collection angle of 44-190 mrad. The samples were cut along the [110] direction of silicon substrates using a FEI Helios 650 dual-beam Focused Ion Beam device. Chemical mapping was performed using electron energy loss spectroscopy (EELS) on a Gatan Quantum ERS spectrometer with a collection angle of 85 mrad, an exposure time of 80 ms/pixel and a 0.5 eV/pixel dispersion in dual EELS mode. Raw data are presented after power-law background subtraction. The EELS fine structures shown in Supplementary Figs. 9 and 15 were processed using Hyperspy by making the sum of the spatially resolved EELS spectra over several SL periods to increase the signal-to-noise ratio and gain a statistical information on the electronic structure changes at the interfaces.

### Second harmonic generation

Light pulses from an amplified Ti:Sapphire laser system at 800 nm with a pulse duration of 45 fs and repetition rate of 1 kHz were converted using an optical parametric amplifier to a probe wavelength of 1200 nm. With this light wavelength, the contribution of the polarization of BaTiO_3_ to the SHG response can be maximized^58,59^. This probe beam was incident onto the sample at 45° with a pulse energy of 15 μJ and a spot size of 250 μm in diameter. The SHG yield was detected using a monochromator set to 600 nm and a photomultiplier tube. The polarization of the incident fundamental wave was set to *α* = 0° or 90°. The emitted frequency-doubled wave was projected through a Glan-Thompson prism as polarization filter at a fixed passing angle *β*. In SHG polarimetry experiments, the polarizer sets the linear polarization of the incident light and the analyzer selects the linear polarization of the frequency-doubled light. Here, we define the 0 degree of the polarizer angle to be vertical and in the up direction to maintain consistency with our previous work^58,59^. We investigate the BTO symmetry using analyzer scans, i.e., by keeping the polarizer angle fixed and rotating the analyzer angle.

### Reporting summary

Further information on research design is available in the Nature Research Reporting Summary linked to this article.

## Supplementary information

Supplementary Information

Editorial Policy Checklist

Reporting Summary

Author checklist

## Data Availability

The data that support the findings of this study are available from the corresponding authors upon reasonable request.
