# Supplementary material for: Signatures of enhanced out-of-plane polarization in asymmetric BaTiO3 superlattices integrated on silicon
Source: Nat Commun. 2022 Jan 11;13:265. doi: 10.1038/s41467-021-27898-x (PMC8752726; doi:10.1038/s41467-021-27898-x)
Supplement: Supplementary file 1 — Supplementary Information [file 41467_2021_27898_MOESM1_ESM.pdf]

Supplementary Information for

Signatures of enhanced out-of-plane polarization in asymmetric $BaTiO_3$ superlattices integrated on silicon

Binbin Chen et al.

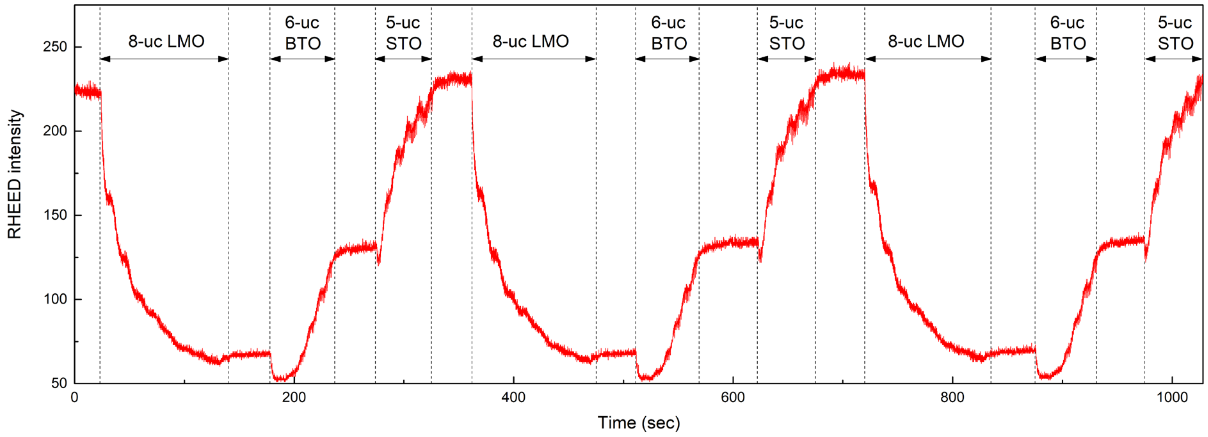

Supplementary Fig. 1 Typical RHEED oscillations of the $L_8B_6S_5$ SL grown on Si/STO.

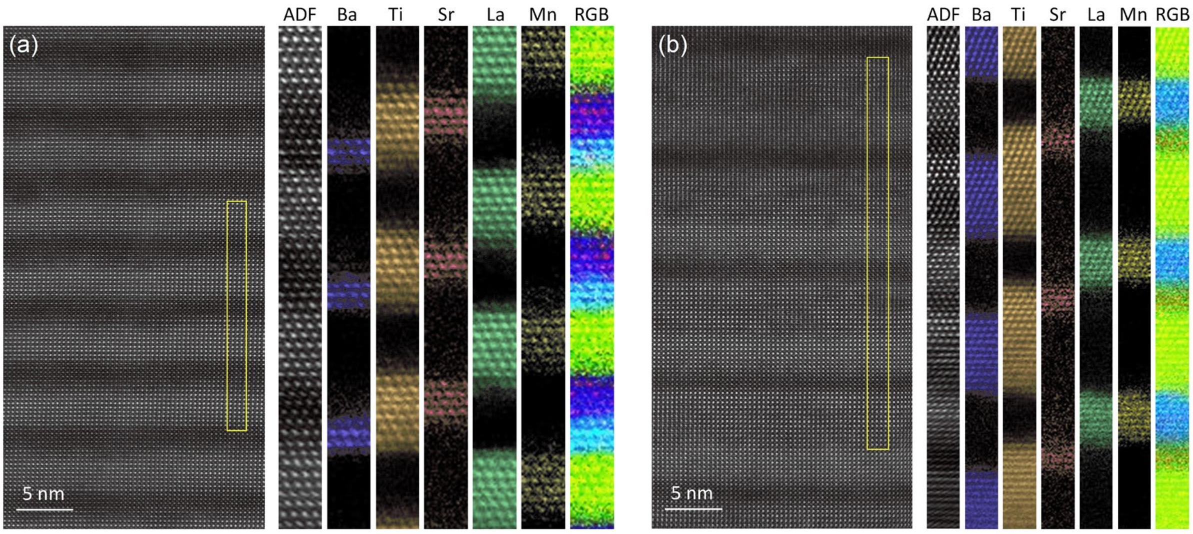

Supplementary Fig. 2 STEM images and EELS elemental maps for the $L_8B_2S_5$ (a) and $L_8B_{12}S_5$ (b) SLs.

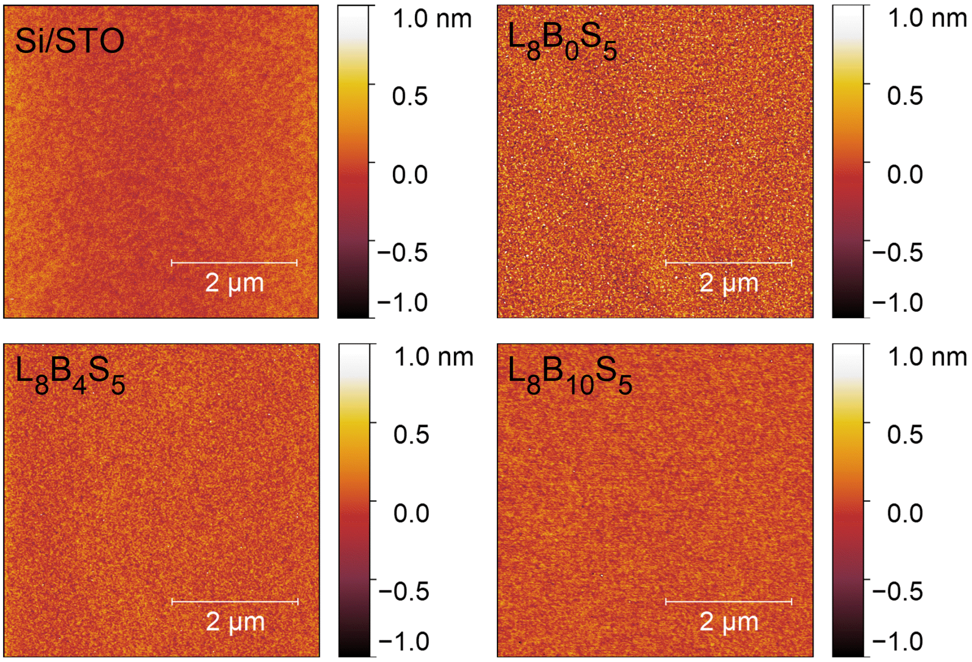

Supplementary Fig. 3 AFM images of the Si/STO substrate and $L_8B_0S_5$, $L_8B_4S_5$ and $L_8B_{10}S_5$ SLs as denoted.

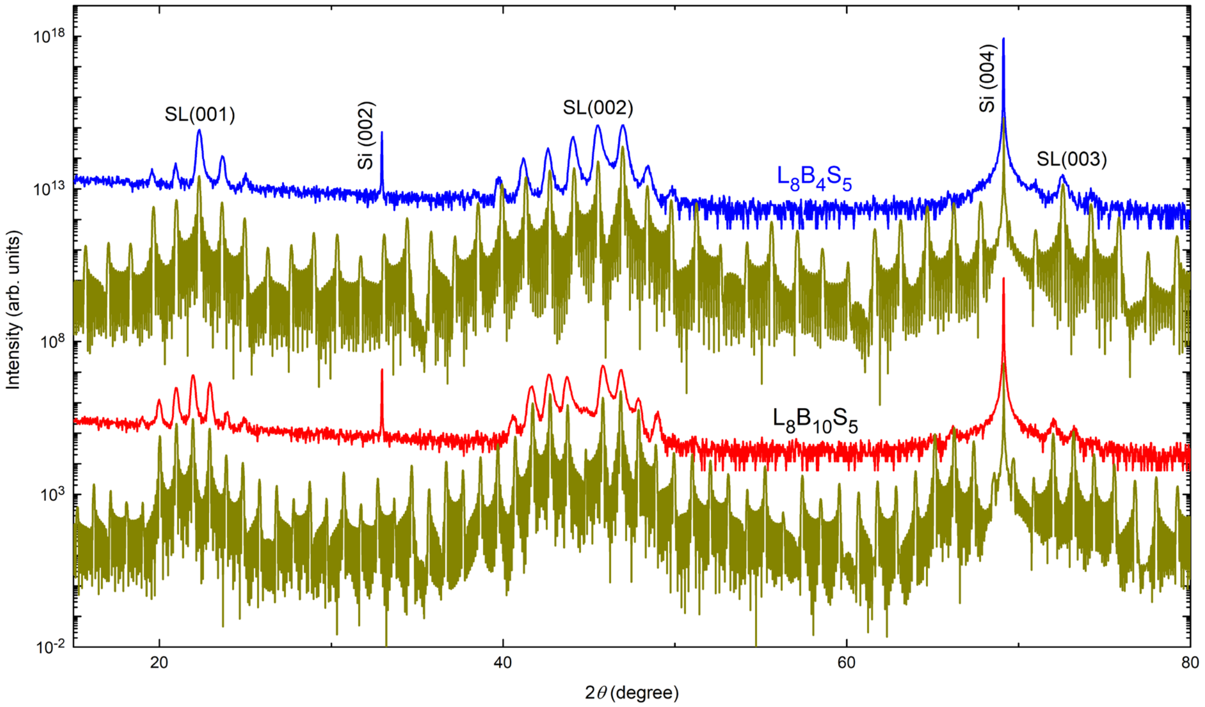

Supplementary Fig. 4 XRD θ-2θ scans of the $L_8B_4S_5$ (blue) and $L_8B_{10}S_5$ (red) SLs, together with simulations using the InteractiveXRDFit program.[1]

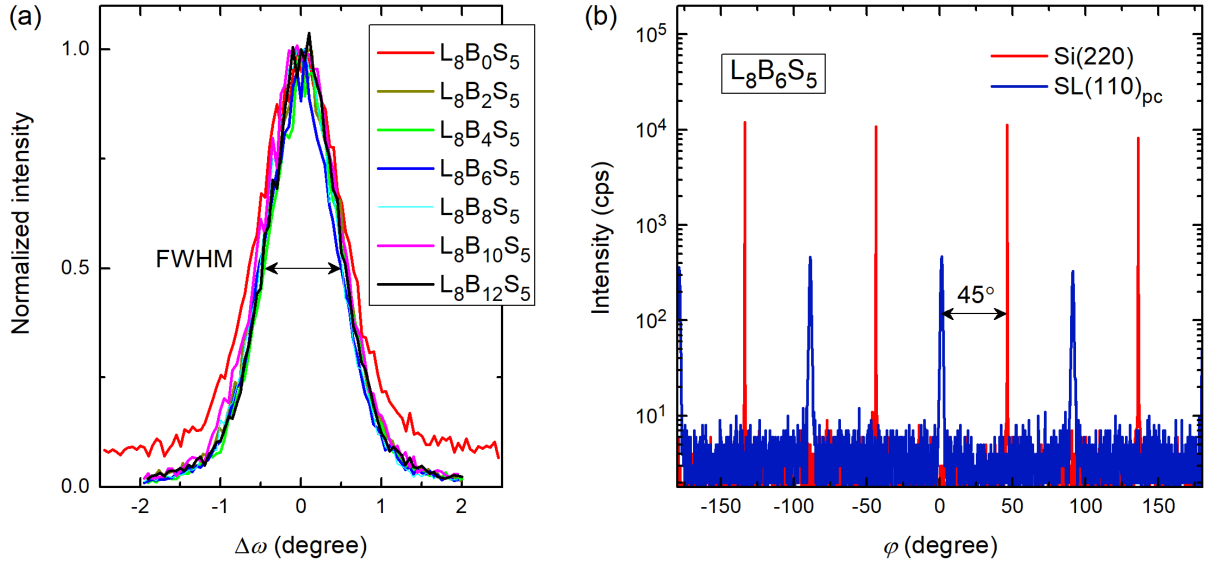

Supplementary Fig. 5 (a) XRD rocking curves around $(001)_{pc}$ peak of LMO/BTO/STO SLs with varied layer thickness of BTO. (b) Phi scans around Si(220) and SL(110)pc reflections measured for the $L_8B_6S_5$ SL.

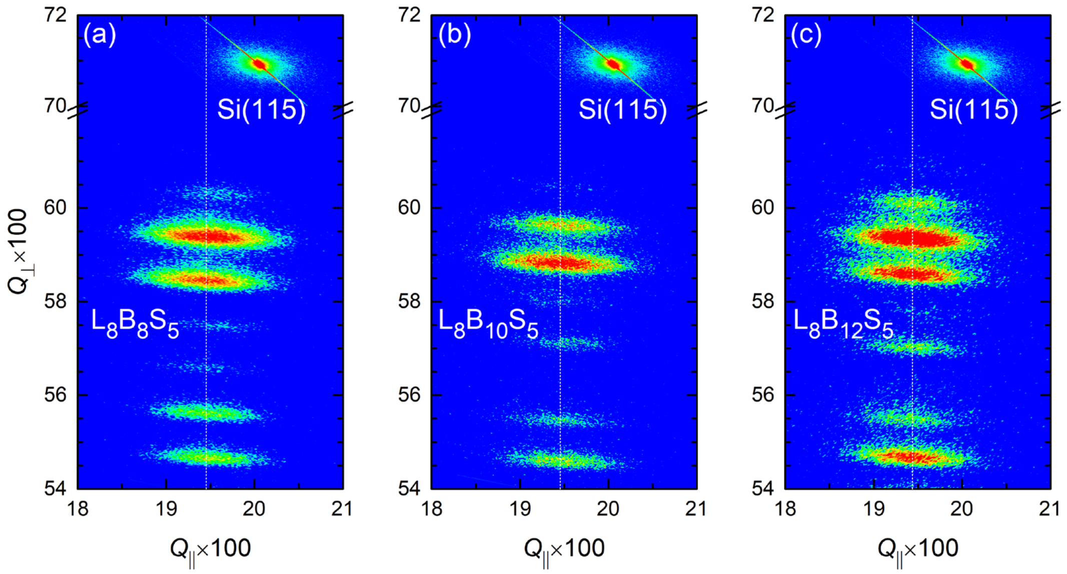

Supplementary Fig. 6 RSMs around the SL $(103)_{pc}$ and Si (115) reflections measured for the $L_8B_8S_5$ (a), $L_8B_{10}S_5$ (b), $L_8B_{12}S_5$ (c) SLs. All SLs are relaxed from the Si substrates as indicated by their different in-plane Q values.

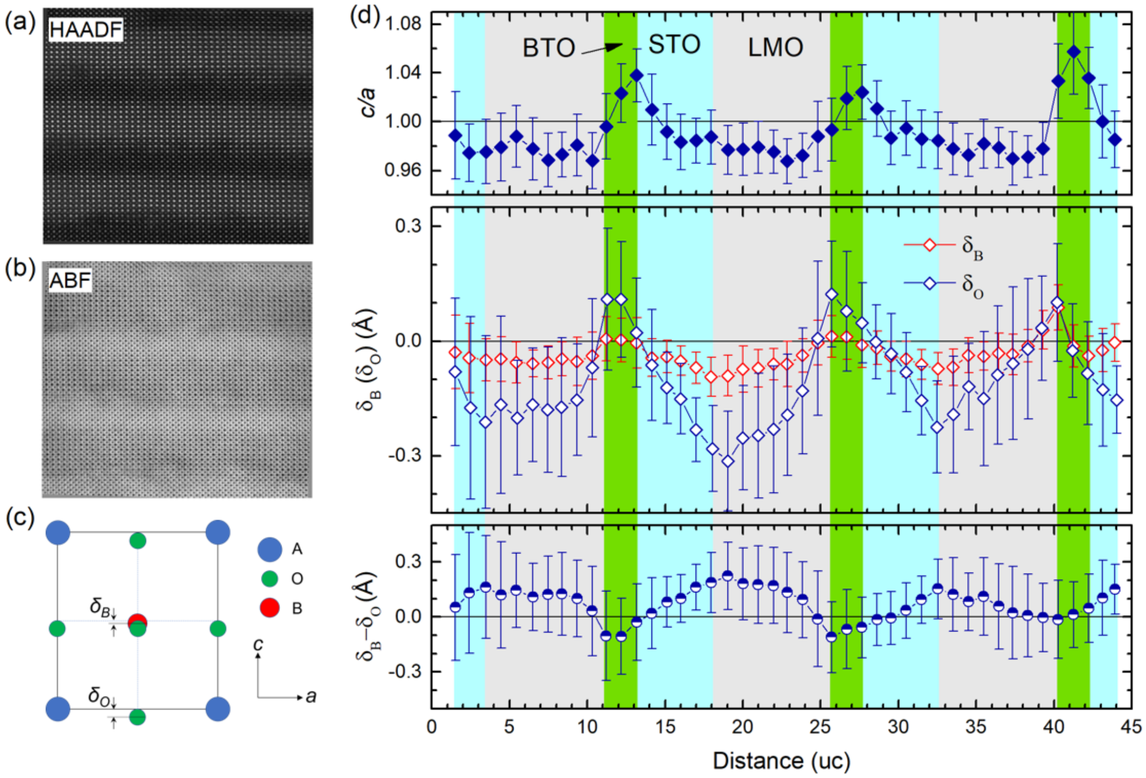

Supplementary Fig. 7 HAADF (a) and ABF-STEM (b) images of the $L_8B_2S_5$ SL. (c) Definition of the B-site ionic displacement δ_B and oxygen displacement δ_O in a ABO_3 perovskite unit cell. δ_B-δ_O characterizes the B-site ionic displacement with respect to oxygen. Positive value means that the ion shifts towards the substrate. (d) Layer dependent tetragonality (*c*/*a*), δ_B (δ_O) and δ_B-δ_O. The error bars show the standard deviation for each atomic plane.

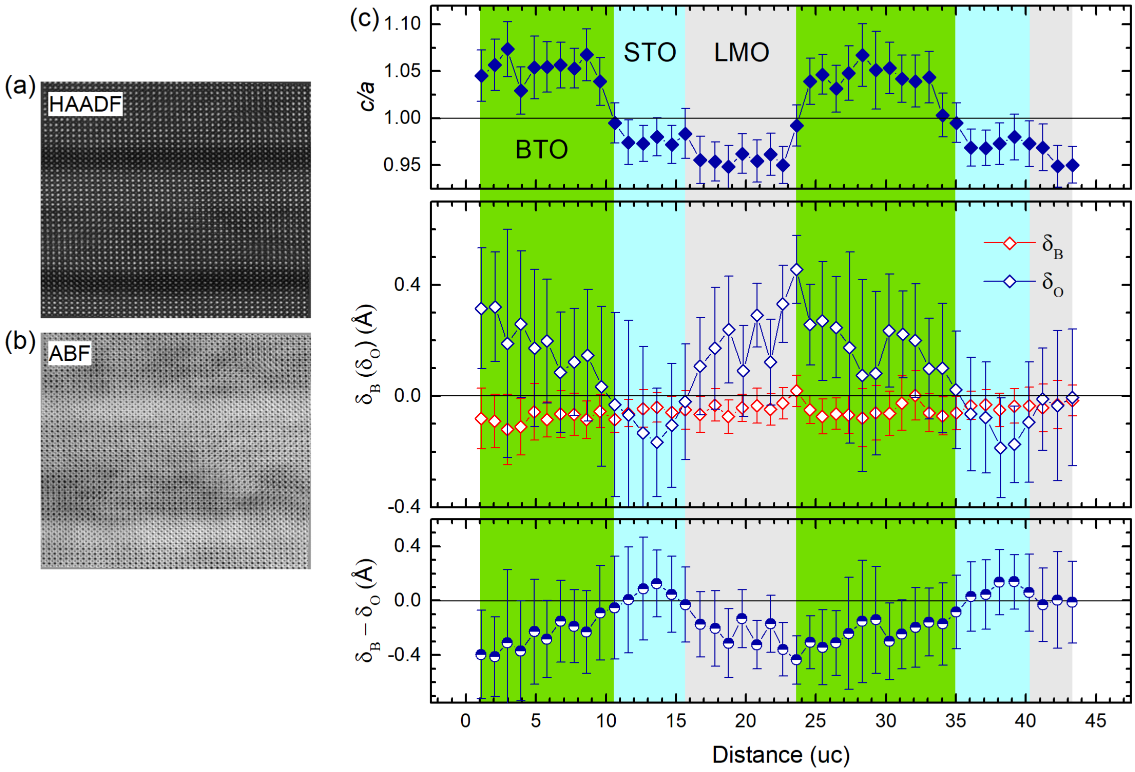

Supplementary Fig. 8 HAADF (a) and ABF-STEM (b) images of the $L_8B_{12}S_5$ SL. (c) Layer dependent tetragonality (*c/a*), δ_B (δ_O) and δ_B-δ_O. The error bars show the standard deviation for each atomic plane. Note that large error bars are included in Supplementary Fig. 7 and Fig. 8 albeit with state-of-the-art STEM instruments and image processing tools. This is mainly because the inherent ionic displacement in BTO is rather small (~10 pm), as compared to Pb-based ferroelectrics.[2] This is approaching the resolution limit of STEM, setting up the main obstacle towards a precise determination of the polarization.[3] Meanwhile, although the superlattices on silicon are of high quality here, they are of course less perfect than those BTO layers grown on perovskite single crystals.[4-6] The sample quality adds extra challenges to our measurements.

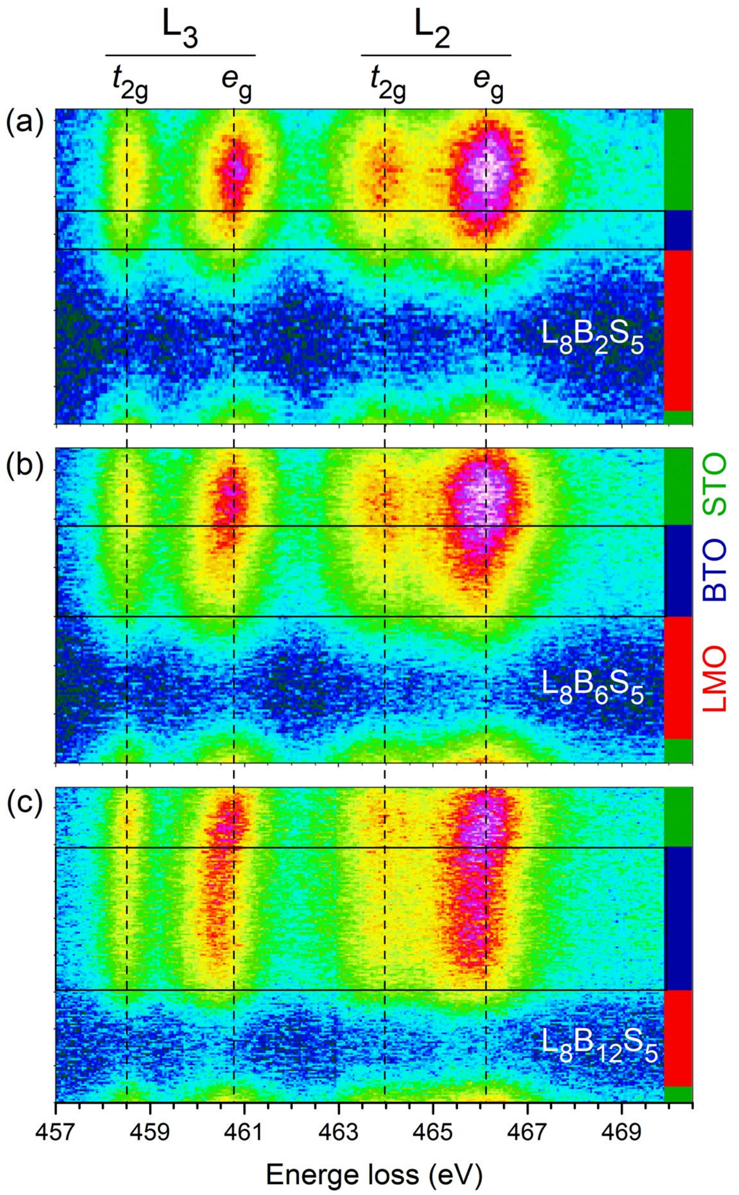

Supplementary Fig. 9 STEM-EELS mapping of the Ti $L_{2,3}$ edges measured in a single SL period for the $L_8B_2S_5$ (a), $L_8B_6S_5$ (b) and $L_8B_{12}S_5$ (c) SLs.

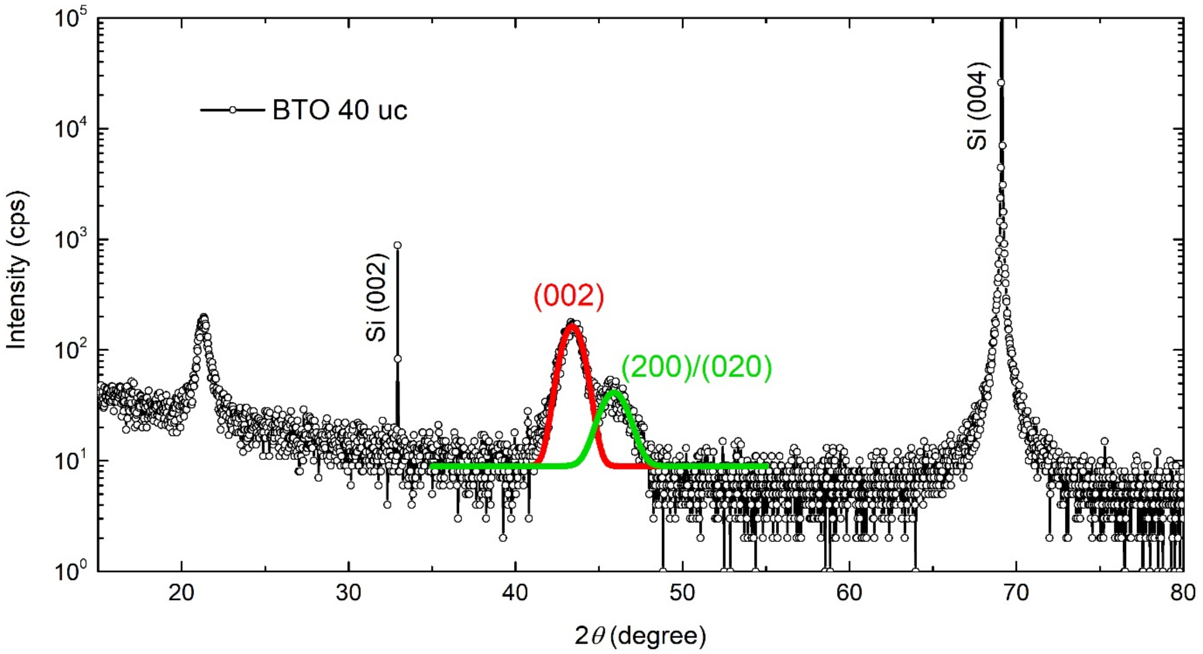

Supplementary Fig. 10 XRD θ-2θ scan of a 40-uc BTO single film grown on Si/STO.

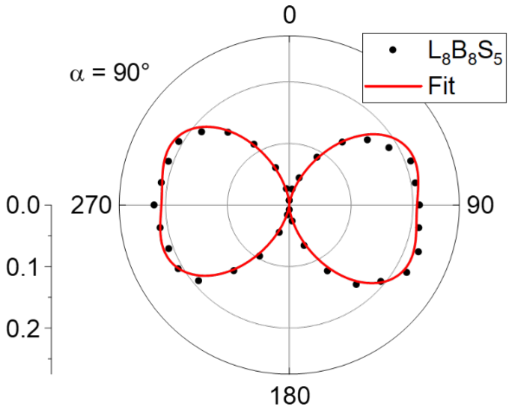

Supplementary Fig. 11 SHG analyzer measurement for the $L_8B_8S_5$ SL on Si/STO at a polarizer angle of α = 90°. The fit was performed for the *4mm* point group according to the procedure described in ref.7.

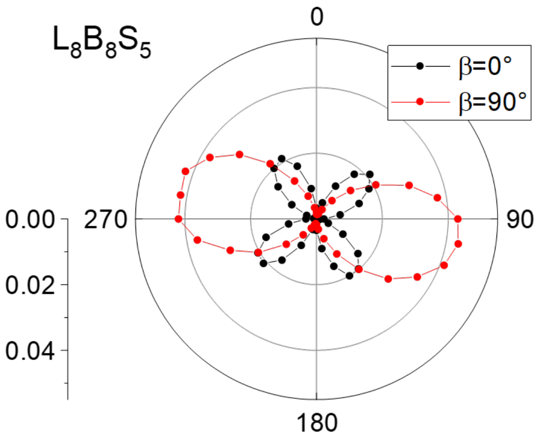

Supplementary Fig. 12 SHG polarizer measurement for the $L_8B_8S_5$ SL on Si/STO at fixed analyzer angles $\beta = 0°$ and $\beta = 90°$.

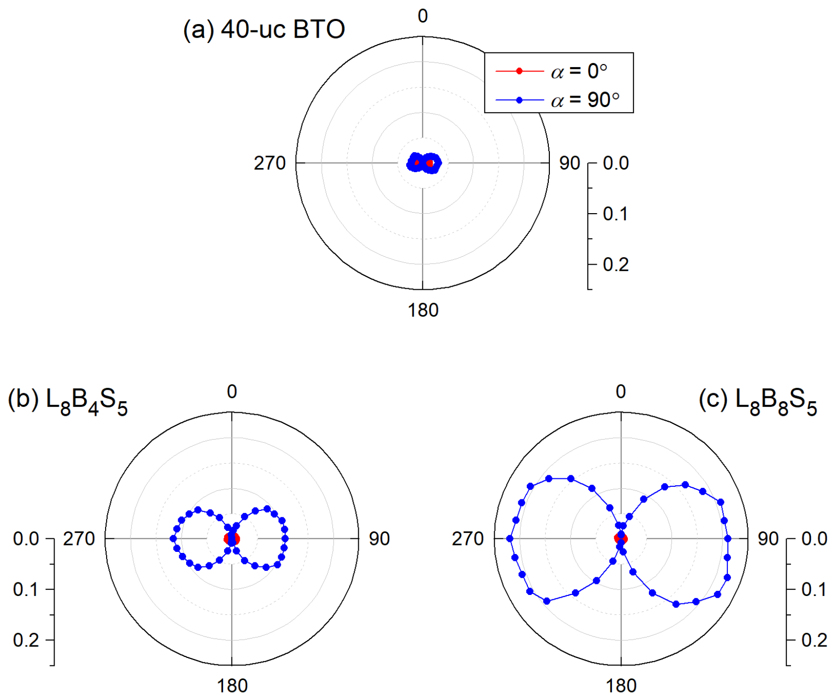

Supplementary Fig. 13 Repeated SHG analyzer measurements for the 40-uc BTO single film (a), $L_8B_4S_5$ (b) and $L_8B_8S_5$ (c) SLs grown on Si/STO at a polarizer angle $\alpha = 0°$ and $\alpha = 90°$. Here, the pulse energy at 1200 nm was set to 20 μJ and the SHG signal was integrated for 2s.

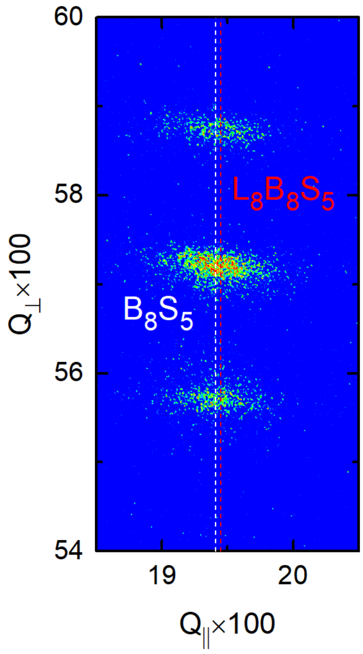

Supplementary Fig. 14 RSM around SL(103)$_{pc}$ reflection of the B_8S_5 SL. The white and red dotted lines indicate the in-plane *Q* values of the B_8S_5 and $L_8B_8S_5$ SLs, respectively.

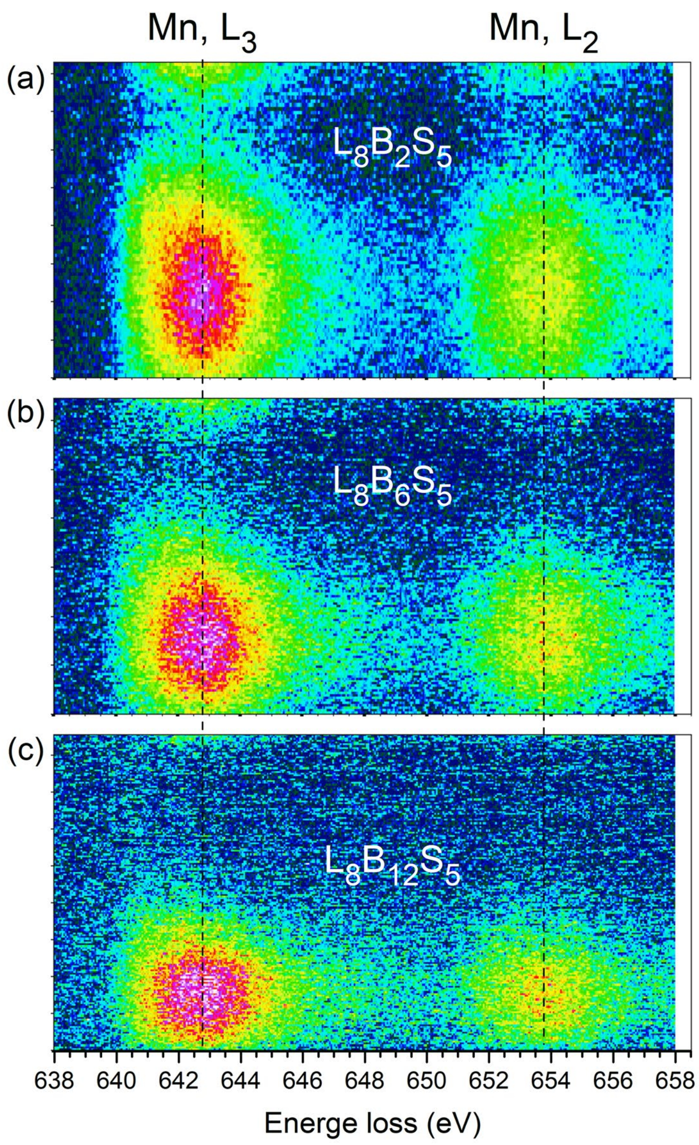

Supplementary Fig. 15 STEM-EELS mapping of the Mn $L_{2,3}$ edges measured in a single SL period for the $L_8B_2S_5$ (a), $L_8B_6S_5$ (b), $L_8B_{12}S_5$ (c) SLs.

Reference:

[1] Lichtensteiger, C. InteractiveXRDFit: a new tool to simulate and fit X-ray diffractograms of oxide thin films and heterostructure. *J. Appl. Cryst.* **51**. 1745-1751 (2018).

[2] Chisholm, M. F. *et al.* Atomic-scale compensation phenomena at polar interfaces. *Phys. Rev. Lett.* **105**, 197602 (2010).

[3] Gauquelin, N. *et al.* Determining oxygen relaxations at an interface: A comparative study between transmission electron microscopy techniques. *Ultramicroscopy*, **181**, 178-190 (2017).

[4] Guo, H. *et al.* Interface-induced multiferroism by design in complex oxide superlattices. *Proc. Natl. Acad. Sci. U. S. A.* **114**, 5062-5069 (2017).

[5] Cao, Y. *et al.* Artificial two-dimensional polar metal at room temperature. *Nat. Commun*. **9**, 1547 (2018).

[6] Wang, L. *et al.* Ferroelectrically tunable magnetic skyrmions in ultrathin oxide heterostructures. *Nat. Mater.* **17**, 1087-1094 (2018).

[7] Denev, S. A. *et al.* Probing ferroelectrics using optical second harmonic generation. *J. Am. Ceram. Soc.,* **94**, 2699-2727 (2011).
