# Supplementary material for: Signatures of enhanced out-of-plane polarization in asymmetric BaTiO3 superlattices integrated on silicon
Source: Nat Commun. 2022 Jan 11;13:265. doi: 10.1038/s41467-021-27898-x (PMC8752726; doi:10.1038/s41467-021-27898-x)
Supplement: Supplementary file 4 — Author checklist [file 41467_2021_27898_MOESM4_ESM.docx]

| 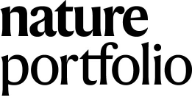 |  |
| --- | --- |
| **Author Checklist** | NCOMMS-NCOMMS-21-15999A |
| **0000000000000000000000000000000000000000000000000000000000000** | **0000000000000000000000000000000000000** |
| **Please check the items below carefully and add a response in each row of the table to indicate the changes that you have made. Please also check through any additional marked-up edits we may have provided within the manuscript file.** |  |
|  |  |
|  |  |
|  |  |
|  |  |
| Author information |  |
| Our guidance: | Your response: |
|  |  |
| Please review your complete author list to verify that it is complete and accurate. We ask that you consult with your coauthors to ensure that all names, affiliations, and titles are represented correctly. Note that if any authors are added or removed after this point then all authors will be requested to provide approval documentation that could potentially delay the production of your paper. | We promoted Morgan Trassin as a corresponding author, and slightly modified the affiliation, as highlighted. |
| Please include a list of the corresponding authors and an e-mail address for each one in the final version of the manuscript file. | We added the information at the bottom of the first page of our manuscript, as highlighted. |
| Please ensure that all affiliations are in the correct sequential order according to their position in the author list. Affiliation 1 must be the first affiliation associated with the first author. Please see this article as an example: https://www.nature.com/articles/s41467-020-16621-x.pdf | Yes |
|  |  |
| Article structure |  |
| Our guidance: | Your response: |
| We can accommodate up to 10 display items (Figures or Tables) in the main article. Each Figure and Table must fit easily within an A4 page (210 x 297 mm). Please ensure that the number and size of your Figures and Tables fulfil these requirements to avoid any delay in the acceptance of your article. | Yes |
| **Please ensure your main manuscript file includes the following sections, in this order:** | Yes |
| *Title Author list Affiliations Abstract Introduction Results Discussion (optional) Results and Discussion (optional) Methods (including Data Availability, Code Availability and Statistics subsections where relevant) References Acknowledgements Author Contributions Statement Competing Interests Statement Tables Figure Legends/Captions (for main text figures)* |  |
| We do not edit Supplementary Information files; they will be uploaded with the published article as they are submitted with the final version of your manuscript. Any tracked changes should be removed from the file and the file should be provided as a PDF file. Supplementary Figures do not need to be provided separately. |  |
|  |  |
|  |  |
| Main text |  |
| Our guidance: | Your response: |
| Please refrain from using words such as new/novel/first, when referring to the scientific findings. | We avoid these words in our manuscript. |
| Please do not use italics, bold font, underlining or speech marks unless required for technical terms (in both the main text and the display items). | Only bold font was used for the section headers (**Results**, **Discussion**, **References**, etc). |
| Please make sure that mathematical terms throughout your manuscript and Supplementary Information (including in figures, figure axes, and legends) conform strictly to the following guidelines. Equations must be supplied in editable format, and not as images. Scalar variables (e.g. x, V, χ) must be typeset in italic, whereas multi-letter variables and functions (e.g. log) must be formatted in roman. Vectors (such as the wavevector k or the magnetic field vector B) must be typeset in bold without italics. | Yes |
|  |  |
| Figures and Tables |  |
| Our guidance: | Your response: |
| Please see the guidelines linked below for detailed instructions about how your figures should be prepared. Following these instructions will reduce the chances of delays should we need to request replacement artwork from you at a later stage. | Yes |
| <https://www.nature.com/documents/NRJs-guide-to-preparing-final-artwork.pdf> |  |
| All colour scales must be defined and intensity levels must be provided in either the figure or its associated legend. Please provide labels to these legends, e.g. at colour bars, in the format “variable [unit]” or similar. | Yes |
| Shadings or symbols in graphs must be defined in some fashion. We prefer that you use a key within the image; do not include colored symbols in the legend/caption. | Yes |
| Please make sure that the terms ‘atomic units (a. u.)’ or ‘arbitrary units (arb. units)’ are appropriately used. | Yes |
| Any abbreviations, symbols or colours present in your figures must be defined in the associated legends. | Yes |
| In each Figure and Supplementary Figure where error bars are used, they must be defined. | Yes |
|  |  |
| Data and Code |  |
| Our guidance: | Your response: |
| Nature journals strongly support public availability of data and code. Please deposit the data and code used in your paper into a public data repository, or alternatively, present the data as Supplementary Information. If data can only be shared on request, please explain why in your Data Availability Statement, and also in the correspondence with your editor.   Please note that for some data types, deposition in a public repository is mandatory. Any restrictions on sharing of these data types must be clearly indicated in the statement and discussed with the editor. More information on our data deposition policies and available repositories can be found here: | The data that support the findings of this study are available from the corresponding authors upon reasonable request. |
| <https://www.nature.com/nature-research/editorial-policies/reporting-standards#availability-of-data> |  |
| All published manuscripts reporting original research in Nature Portfolio journals must include a data availability statement, as a separate section before the References and under the heading 'Data Availability'.   The data availability statement must make the conditions of access to the “minimum dataset” that are necessary to interpret, verify and extend the research in the article, transparent to readers.   This minimum dataset may be provided through deposition in public community/discipline-specific repositories, custom proprietary repositories or general repositories like Figshare, Zenodo and Dryad. Providing large datasets in supplementary information is strongly discouraged and the preferred approach is to make data available in repositories. Scientific Data, a Nature Portfolio journal, maintains a list of approved and recommended data repositories to support researchers seeking suitable repositories for their data (https://www.nature.com/sdata/policies/repositories).  The Data Availability Statement should also reference any source data published alongside the paper.  If DOIs are provided, we also strongly encourage including these in the Reference list (authors, title, publisher (repository name), identifier, year).  For clinical datasets or third party data, please ensure that the statement adheres to our policy (https://www.nature.com/nature-research/editorial-policies/reporting-standards#availability-of-data) | We provided a separate section of “Data Availability” in our manuscript. |
| Please use the following template to provide all the information stated above:  The XX data generated in this study have been deposited in the YY database under accession code ZZ [add hyperlink here]. The XX data are available under restricted access for {insert reason}, access can be obtained by {explain how}. The raw XX data are protected and are not available due to data privacy laws. The processed XX data are available at YY. The XX data generated in this study are provided in the Supplementary Information/Source Data file. The XX data used in this study are available in the YY database under accession code ZZ [Add hyperlink here]. | Yes |
| An updated lasing reporting summary must be completed and uploaded as a supplementary information file with the revised manuscript. All points on the reporting summary must be addressed; if needed, please revise your manuscript in response to these points. This checklist is published alongside your manuscript online. Please note that this form is a dynamic "smart pdf" and must therefore be downloaded and completed in Adobe Reader, instead of opening it in a web browser. https://www.nature.com/authors/policies/Lasers.pdf | We prepared an updated lasing reporting summary and submitted as a supplementary information file. |
|  |  |
|  |  |
| References |  |
| Our guidance: | Your response: |
| Supplementary References should appear at the end of the Supplementary Information file, and must be self-contained and numbered from 1. References mentioned in both the main text and the Supplementary Information should be part of both reference lists so that the Supplementary Information does not refer to the reference list in the main paper and vice versa. | We confirm that the reference lists are independent in our main paper and Supplementary Information. |
|  |  |
| End matter |  |
| Our guidance: | Your response: |
| The Competing Interests statement must encompass all authors. Please edit the statement accordingly (such as by adding 'The remaining authors declare no competing interests', if this is accurate). | Our Competing Interests statement has been modified by including “The remaining authors declare no competing interests”, as highlighted. |
| Nature Portfolio defines Competing Interest (CI) as financial and non-financial interests (including but not limited to funding, employment, stocks, shares, patents, personal or professional relationships with individuals or institutions, and unpaid membership advocacy) that could be perceived to directly undermine the objectivity, integrity, and value of a publication, or could be seen as having an influence on the judgments and actions of authors with regard to objective data presentation, analysis, and interpretation.  Please thoroughly review our policy on Competing Interests and include a detailed statement both in your final manuscript file and in our manuscript tracking system. Please ensure the statements are identical in both. Be specific about how each point stated relates to the research and list applicable author initials, and/or patent numbers.  If there are no competing interests, a negative statement must be included. | Yes |
| <https://www.nature.com/nature-research/editorial-policies/competing-interests> |  |
| Please confirm that all relevant funding awarded to each author is described in the Acknowledgements section. List each grant number, followed by the initials of the author who received it. | Yes |
|  |  |
| Preparing your manuscript files |  |
| Our guidance: | Your response: |
| Unless otherwise stated please limit individual file sizes to approximately 30MB. We strongly encourage the use of repositories for large datasets or source data due to size considerations. | Yes |
| Please supply a brief (maximum 250 characters, including spaces) summary of the main findings of the paper to be used on our website and in our e-alerts. The summary should be written in the third person in language suitable for a broad audience. The summary may be edited by the editors prior to publication. Please provide this summary in your cover letter.  Format of brief summary:  Two sentences, includes one sentence background. The second sentence begins with ‘Here, the authors…’ | A brief summary is included in our cover letter. |
| Please provide figures as individual vector files with editable text. Acceptable file types for figures are .ai, .eps, .pdf or Chem Draw for fully editable vector-based art. For detailed guidance on figure preparation, see https://www.nature.com/documents/aj-artworkguidelines.pdf | Yes, all figures in the main text are provided as individual PDF files. |
| Please supply the main Supplementary Information file as a single PDF file. | Yes |
| The use or adaptation of previously published images is strongly discouraged. If this is unavoidable, please request the necessary rights documentation to re-use such material from the relevant copyright holders and return this to us when you submit your revised manuscript. Please check whether your manuscript or Supplementary Information contain third-party images, such as figures from the literature, stock photos, clip art or commercial satellite and map data.  For more information on what constitutes ownership by a third party, please contact our Editorial Assistant at naturecommunications@nature.com | We have not used any previously published images or third-party images in our manuscript. |
|  |  |
|  |  |
| Forms to complete |  |
| Our guidance: | Your response: |
| **Editorial Policy Checklist** |  |
| Please update and upload a final version of the Editorial Policy Checklist with your revised manuscript files. A blank Editorial Policy Checklist can be found via the link below. Note that this form is a dynamic ‘smart pdf’ and must be downloaded and completed in Adobe Reader. | A final version of the Editorial Policy Checklist has been submitted with our revised manuscript. |
| Please update your current checklist or download from: |  |
| <https://www.nature.com/documents/nr-editorial-policy-checklist.zip> |  |
|  |  |
|  |  |
| **You will need to upload:** |  |
| Editorial Policy Checklist | Yes |
| Completed Third Party Rights Table (if relevant) | No |
| A point-by-point response to the reviewers' comments | Yes |
| A completed copy of this checklist | Yes |
| The main article file in Microsoft Word format - please supply a version with tracked changes and a version with tracked changes accepted | Yes |
| Separate Figure files | Yes |
| Inventory of Supporting Information | Yes |
| A Supplementary Information file | Yes |
| Lasing Reporting Summary | Yes |
|  |  |
